# Nasal Reflexes

**Published:** 1897-03-20

**Authors:** 


### NASAL REFLEXES.

In the last of the Lettsomian lectures, delivered before the Medical Society, Dr. de Havilland Hall drew attention to the relationship which was found between irritable conditions of the nasal mucous membrane and states of hysteria, neurasthenia, and melancholia. There had been, he thought, a tendency of late years to exaggerate the effect of various intra-nasal lesions. Though he had been examining the interior of noses for the last twenty years, and had constantly used the probe, he had never succeeded in thereby starting an attack of hysteria, although it had been stated that irritation of certain parts of the mucous membrane, the so-called hystero-genetic zones, would produce such attacks, which had ceased to occur after application of the galvano cautery to these areas. He also stated that although in neurasthenic subjects certain abnormal conditions of the mucous membrane would give rise to symptoms of a reflex character which were curable by local treatment, the cure of the condition complained of by no means involved the cure of the patient; and he instanced the case of a patient who had undergone many things for the relief of paroxysmal sneezing, with which she had been affected for many years. She certainly lost her sneezing, but since it disappeared she had suffered from flatulent dyspepsia, acidity, and intestinal catarrh, accompanied by neuralgia and various sensations referred to the pelvic viscera. She had lost flesh, was terribly depressed, and was a typical example of neurasthenia. Doubtless the operations she had undergone had relieved the sneezing, but she dated all the symptoms from which she then suffered to the time when she ceased to sneeze! To relieve the particular symptom complained of is but a small step, perhaps not a step at all, in relieving the condition of neurasthenia on which the various symptoms depend.

